# The effect of an online exercise programme on bone health in paediatric cancer survivors (iBoneFIT): study protocol of a multi-centre randomized controlled trial

**DOI:** 10.1186/s12889-020-09607-3

**Published:** 2020-10-08

**Authors:** Jose J. Gil-Cosano, Esther Ubago-Guisado, Maria J. Sánchez, Maria J. Ortega-Acosta, Maria E. Mateos, Ana I. Benito-Bernal, Francisco J. Llorente-Cantarero, Francisco B. Ortega, Jonatan R. Ruiz, Idoia Labayen, Vicente Martinez-Vizcaino, Dimitris Vlachopoulos, Manuel Arroyo-Morales, Manuel Muñoz-Torres, Juan F. Pascual-Gázquez, Maria C. Vicho-González, Luis Gracia-Marco

**Affiliations:** 1 PROFITH “PROmoting FITness and Health through Physical Activity” Research Group, Sport and Health University Research Institute (iMUDS), Department of Physical Education and Sports, Faculty of Sport Sciences, University of Granada, 18071 Granada, Spain; 2 Andalusian School of Health (EASP), Granada, Spain; 3 Instituto de Investigación Biosanitaria Ibs.GRANADA, Granada, Spain; 4 CIBER of Epidemiology and Public Health (CIBERESP), Madrid, Spain; 5 Department of Preventive Medicine and Public Health, University of Granada, Granada, Spain; 6 Servicio de Pediatría y Oncohematología Pediátricas, Hospital Universitario Virgen de las Nieves, Granada, Spain; 7 Pediatric Oncology Unit, Department of Pediatrics, Reina Sofia University Hospital, Córdoba, Spain; 8 Maimonides Institute for Research in Biomedicine of Cordoba (IMIBIC), Córdoba, Spain; 9 Hospital Infantil Universitario Niño Jesús, Madrid, Spain; 10 CIBEROBN, (Physiopathology of Obesity and Nutrition) Institute of Health Carlos III (ISCIII), 28029 Madrid, Spain; 11 Department of Specific Didactics, Faculty of Education, University of Córdoba, 14071 Córdoba, Spain; 12 Institute for Innovation and Sustainable Development in Food Chain (IS-FOOD), Navarra’s Health Research Institute (IdiSNA), Department of Health Sciences, Public University of Navarra, Calle Tajonar 22, 31006 Pamplona, Navarra Spain; 13 Universidad de Castilla-La Mancha, Health and Social Research Center, Cuenca, Spain; 14 Faculty of Health Sciences, Universidad Autónoma de Chile, Talca, Chile; 15 Children’s Health and Exercise Research Centre, Sport and Health Sciences, University of Exeter, Exeter, UK; 16 Biohealth Research Institute in Granada (ibs.GRANADA), E-18012 Granada, Spain; 17 Department of Physiotherapy, University of Granada, E-18016 Granada, Spain; 18 ”Cuídate” Support Unit for Oncology Patients (UAPO), Sport and Health University Research Institute (iMUDS), E-18016 Granada, Spain; 19 Bone Metabolic Unit, Endocrinology and Nutrition Division, Hospital Universitario San Cecilio, Instituto de Investigación Biosanitaria de Granada (Ibs.GRANADA), Granada, Spain; 20 CIBERFES, Instituto de Salud Carlos III, Madrid, Spain; 21 Department of Medicine, Universidad de Granada, Granada, Spain

**Keywords:** Telemedicine, Cancer, Survivor, Bone, Plyometric exercise, Paediatrics, Quality of life

## Abstract

**Background:**

New approaches on paediatric cancer treatment aim to maintain long-term health. As a result of radiotherapy, chemotherapy or surgery, paediatric cancer survivors tend to suffer from any chronic health condition. Endocrine dysfunction represents one of the most common issues and affects bone health. Exercise is key for bone mass accrual during growth, specifically plyometric jump training. The iBoneFIT study will investigate the effect of a 9-month online exercise programme on bone health in paediatric cancer survivors. This study will also examine the effect of the intervention on body composition, physical fitness, physical activity, calcium intake, vitamin D, blood samples quality of life and mental health.

**Methods:**

A minimum of 116 participants aged 6 to 18 years will be randomized into an intervention (*n* = 58) or control group (*n* = 58). The intervention group will receive an online exercise programme and diet counselling on calcium and vitamin D. In addition, five behaviour change techniques and a gamification design will be implemented in order to increase the interest of this non-game programme. The control group will only receive diet counselling. Participants will be assessed on 3 occasions: 1) at baseline; 2) after the 9 months of the intervention; 3) 4 months following the intervention. The primary outcome will be determined by dual energy X-ray absorptiometry (DXA) and the hip structural analysis, trabecular bone score and 3D-DXA softwares. Secondary outcomes will include anthropometry, body composition, physical fitness, physical activity, calcium and vitamin D intake, blood samples, quality of life and mental health.

**Discussion:**

Whether a simple, feasible and short in duration exercise programme can improve bone health has not been examined in paediatric cancer survivors. This article describes the design, rationale and methods of a study intended to test the effect of a rigorous online exercise programme on bone health in paediatric cancer survivors. If successful, the iBoneFIT study will contribute to decrease chronic health conditions in this population and will have a positive impact in the society.

**Trial registration:**

Prospectively registered in isrctn.com: isrctn61195625. Registered 2 April 2020.

## Background

Owing to major advances in cancer screening and treatment over the last 30 years, cancer survival has improved dramatically. In Europe, the paediatric cancer incidence increases annually by 0.54% in children (0–14 years) and by 0.96% in adolescents (15–19 years), although it seems that the incidence in adolescents is decelerating [1]. Nevertheless, the 5-year survival rate is now at 77.9% for children and 79% for adolescents and young adults (20–39 years) [2, 3]. Unfortunately, the treatment of paediatric cancer by means of radiation, chemotherapy and/or surgery is associated with various late effects (e.g. impaired growth, musculoskeletal sequelae, cardiopulmonary compromise and secondary malignancy) [4–6], predisposing paediatric cancer survivors to disabling conditions [4]. Furthermore, paediatric cancer treatment has been documented to have an effect on emotional well-being and quality of life, with survivors reporting anxiety, depression and post-traumatic stress [7, 8].

Paediatric cancer is a life-threatening condition that also occurs during the period of bone development and strengthening. Gonadal failure following to pelvic radiation or gonadotoxic chemotherapy and hypothalamic pituitary dysfunction by means of cranial radiation can adversely affect areal bone mineral density (aBMD), increasing osteoporosis risk later in life [9, 10]. In addition, direct radiation to bone not only causes hypovascularity but has a direct cytotoxic effect on the epiphyseal chondrocytes [11]. Observational studies have found low aBMD during and after cancer treatment to be associated with increased fracture risk (80% increase for every 1 SD reduction in lumbar spine aBMD Z-score) [12–15], which can lead to a higher risk of osteopenia and osteoporosis in adulthood and finally, disability [16, 17]. Moreover, data from a review showed that up to 68% of paediatric cancer survivors presented moderate-to-severe aBMD deficits (Z-score < − 1), while up to 46% had severe aBMD deficits (Z-score < − 2) [18].

The attainment of peak bone mass during childhood and adolescence determines the aBMD later in life and therefore the onset of osteoporosis [19, 20]. This process has a strong genetic component, although lifestyle factors (i.e. physical activity and dietary habits) contribute up to 20% of the variation in peak bone mass [21]. High-intensity, weight bearing physical activity that elicit a variety of strains and include multiple rest periods is known to improve bone mass [22–25], accrual [26] and maintenance [27] as the skeleton adapts to the loads under which it is placed. Likewise, an adequate calcium and vitamin D intake in combination with physical activity is necessary to obtain beneficial gains in bone health in children and adolescents [28, 29]. In this sense, calcium and vitamin D supplementation did not add benefit to nutritional counselling for improving bone outcomes among adolescents and young adults survivors of acute lymphoblastic leukaemia [30].

### Exercise and bone health

Exercise contributes to the development of bone mass in youths due to its association with increases in lean mass [31, 32]. Larger muscles exert greater forces on the bones, which will adapt and therefore improve their strength [33]. Furthermore, plyometric jump training is one of the best methods to improve bone health since the impacts produced against the ground will cause higher forces on the bones [34]. A recent systematic review has shown that plyometric jump training causes improvements in bone mineral content (BMC), aBMD and structural properties in children and adolescents [35]. More specifically, an 8-month jumping intervention (~ 3 min/day) improved bone mass in the proximal femur in pubertal children [36]. Mackelvie et al. [37] showed that a 7-month jumping intervention (10 min, 3 times/week) enhanced bone mass in the femoral neck and lumbar spine in pubertal girls. Additionally, Vlachopoulos et al. [38, 39] found that a 9-month jumping intervention (10 min, 3 to 4 times/week) improved bone outcomes in adolescent males participating in non-osteogenic sports and with poorer bone health.

A similar effect might be seen in survivors of paediatric cancer. A randomized controlled trial (RCT) in children with acute lymphoblastic leukaemia showed that resistance exercise was unsuccessful in preventing the reduction in aBMD [40]. However, the intervention (duration, load) was not properly described. A RCT focusing on low-magnitude, high frequency mechanical stimulation seemed to improve total body aBMD in paediatric cancer survivors, while a reduction was observed in the placebo group [41]. In a recent study in children with cancer, the exercise programme was not successful in improving aBMD nor other factors such as physical function or health-related quality of life [42]. This was because exercise requires of certain intensity to modify these factors and this could not be achieved during treatment due to the child’s responses to the treatment and disease. Considering the gap in the literature, and taking into account the Exercise Guidelines for Cancer Survivors [43, 44], it is crucial to develop and implement feasible exercise programmes focused on improving bone health into survivorship.

The aim of this study is to investigate the effect of a 9-month online exercise programme on bone health in paediatric cancer survivors aged 6–18 years and to follow up these outcomes 4 months after the intervention to determine the extent of residual effect. We hypothesize that the intervention stimulus will be enough to improve bone health in this population. We will also examine the effect of the intervention on body composition, physical fitness, physical activity, calcium intake, vitamin D, blood samples quality of life and mental health.

## Methods/ design

### Study design

**Fig. 1:**
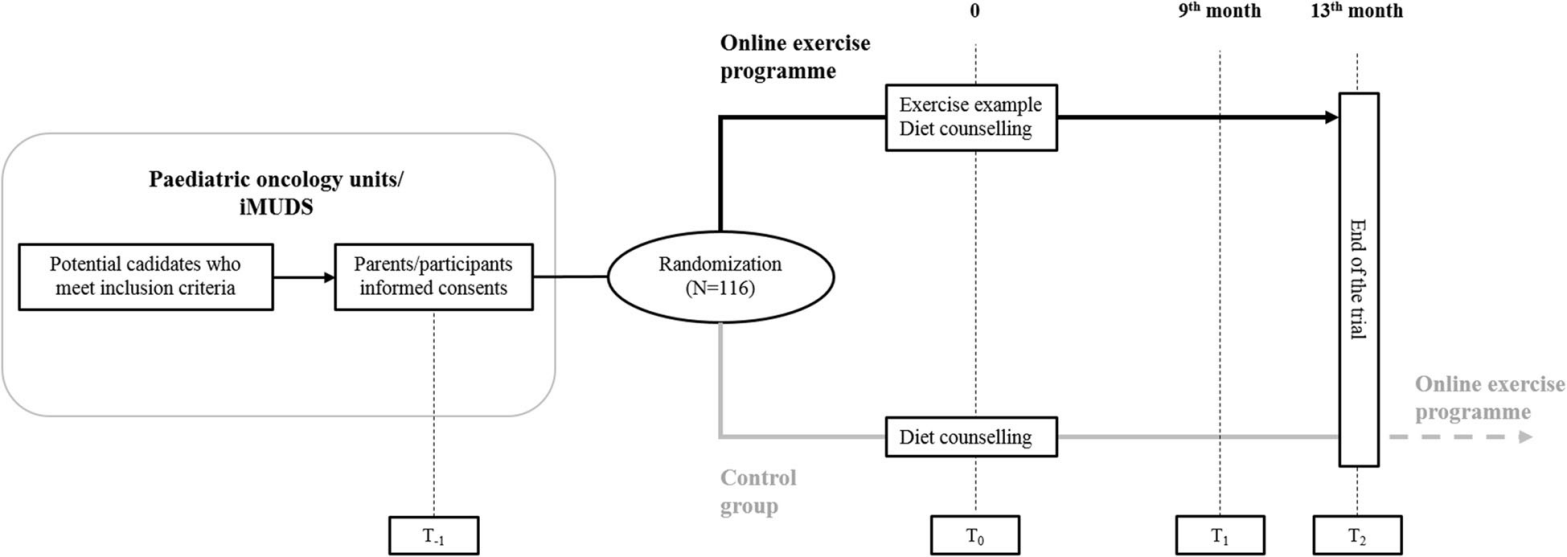
iBoneFIT study design. T_− 1_, meeting with parents and participants; T_0_, baseline assessment; T_1_, post-intervention assessment; T_2_, follow-up assessment. iMUDS *Sport and Health University Research Institute*

This protocol is reported based on Standard Protocol Items: Recommendations for Interventional Trials (SPIRIT) guidelines [45]. The iBoneFIT study is a multi-centre, parallel groups RCT (1:1) designed under the equivalence basis and registered in isrctn.com (Reference: isrctn61195625, 2 April 2020). Eligible participants from two paediatric oncology units of Southern Spain will be contacted, informed, and if consenting, enrolled into the study after a meeting (T_− 1_) (see recruitment section). Then, randomization will be performed by an external partner who is independent of the participant recruitment and enrolment process (see randomization section). Assessments will be conducted at baseline (T_0_) and after nine (T_1_) and thirteen (T_2_) months in the Sport and Health University Research Institute (iMUDS, University of Granada). After finishing the study, participants in the control group will be offered the same online exercise programme. A graphical description of the study design is shown in Fig. 1.

### Ethical approval

The study will be performed following the ethical guidelines of the Declaration of Helsinki, last modified in 2013. This study has been checked and approved by the Ethics Committee on Human Research of Regional Government of Andalusia (Reference: 4500, December 2019).

### Inclusion and exclusion criteria

The iBoneFIT study will include paediatric cancer survivors: 1) aged 6 to 18 years; 2) diagnosed at least 1 year earlier; 3) to have been exposed to radiotherapy and/or chemotherapy; and 4) not currently receiving treatment for cancer.

Exclusion criteria are defined as follows: 1) simultaneous participation in another study that place participants at any additional risk, discomfort or affect the results of both studies; 2) previous diagnosed anorexia nervosa/bulimia, known pregnancy and/or known alcohol and drug abuse; 3) children requiring chronic oral glucocorticoid therapy; 4) having an injury that may affect daily life activities and can be aggravated by exercise; and 5) to have a lower limb prosthesis that prevent bone assessment.

### Recruitment

Eligible participants will be contacted via telephone calls or information letters from the Units of Paediatric Oncology of the ‘Virgen de las Nieves’ (Granada) and ‘Reina Sofía’ (Córdoba) University Hospitals in Southern Spain. A short study information brochure will be used in routine check-ups. A meeting will be held with potential participants and parents/tutors to carefully inform about the benefits and risks of the study, and researchers will answer any question that they may have. Then, informed consents will be given, and participants will have 15 days to send it to the researchers. A hotline will be available to clarify remaining questions about the study. Those who do not react to the study invitation will be followed up via phone call at the end of these 15 days in order to check if they wish to participate. All participants will sign the informed consent before their visit to the iMUDS.

### Randomization and blinding

Randomization to an intervention group (online exercise programme, IG) or control group (no treatment, CG) will be performed by an external partner (V.M-V) who is independent of the participant recruitment and enrolment process, stratified by age and sex. Each participant will be provided a uniform (0, 1) random number using SAS software, version 9.1 (SAS Institute Inc), within their respective age and sex group. Assignments will be blinded to the assessors until all tests are completed. For feasibility reasons, the study will probably be conducted in two waves of 58 children at most.

### Sample size

We have used femoral neck aBMD as the outcome to calculate the sample size, since it is a key variable in the diagnosis of osteoporosis. Since the study will include children and adolescents (6–18 years), the sample size has been calculated taking into account that sub-group analysis by age groups (6 to 11 years and 12 to 18 years) may be required. Based on an expected effect size of 0.25 for the change in femoral neck aBMD, an α level of 0.05 and a power of 80%, a minimum of 116 participants will be required (IG = 58 and CG = 58). This includes a 20% extra for occasional losses and refusals and 10% for multivariable analyses. Calculations have been obtained using G*Power (v.3.1.9.2) with analysis of variance: repeated measures (within-between interactions) for 2 groups (between factors) and 2 time points (pre, post, within factors). A correlation between measures of 0.7 has been assumed, which is achievable when measuring bone outcomes [16].

### Statistical analysis

All variables will be checked for normality using both statistical and graphical methods. Results will be presented as frequencies and proportions with 95% confidence intervals for categorical variables and mean (standard deviation) or median (range) for continuous variables. A descriptive analysis of the participants characteristics will be performed as soon as the baseline assessments are completed. This cross-sectional analysis will show the comparability of IG and CG and the need for adjustment when between group comparisons are done.

General Lineal Models will be used to examine the training effects [time (pre-post 9-month intervention) x group interaction] on the primary and secondary outcomes. Change in bone outcomes will be used as age and sex-adjusted z-scores. The baseline level of each outcome variable will be entered as covariate. Effect sizes will be reported. Multiple imputation methods and sensitivity analysis (i.e. propensity score) will be performed to handle missing data and appreciate the potential influence of missing responses. Finally, in the event of possible dropouts, a statistical analysis will be carried out by protocol and intention to treat.

### Participant adherence and compliance

Participants will be allowed to withdraw at any time; nevertheless, several strategies will be used for adherence and compliance with the intervention. The minimum compliance allowed at each phase of the intervention will be 50% but the overall compliance after 9 months will have to reach 70%. A lack of compliance (< 50%) without justified reasons in the first phase of the intervention will result in the participant being invited to drop out from the study. This 70% adherence rate means completing 95 sessions of 136. If a participant has not completed 70% of the intervention by the end of the 9 months but can reach 70% within two additional weeks, the exercise programme will be extended for them. Compliance with the intervention will be monitored using a diary and it will be sent to the research staff on a monthly basis (Item 5). Parental involvement will be requested for this matter.

Participants and their parents are verbally motivated to participate in the intervention and to attend to all the assessments. Children who complete successfully the intervention will get a certificate of achievement. Children are the key part of this study and they deserve acknowledgements for their positive attitude and willingness (and their family) to participate in this study.

### Intervention

#### Exercise programme rationale

The rationale of the iBoneFIT exercise programme will be described following the Consensus on Exercise Reporting Template (CERT) criteria recommendations [46]. The items detailing the recommendations are shown in Table 1.

**Table 1:** CERT checklist from iBoneFIT study exercise programme

Item	Checklist item	Identification (section)
1	Detailed description of exercise equipment	Exercise programme characteristics
2	Detailed description of the qualifications, expertise and/or training	Exercise programme characteristics
3	Describe whether exercises are performed individually or in a group	Exercise programme characteristics
4	Describe whether exercises are supervised or unsupervised; how they are delivered	Exercise programme characteristics
5	Detailed description of how adherence to exercise is measured and reported	Participant adherence and compliance
6	Detailed description of motivation strategies	Exercise programme characteristics
7a	Detailed description of the description rule(s) determining exercise progression	Exercise programme rationale
7b	Detailed description of how the exercise programme was progressed	Periodisation
8	Detailed description of each exercise to enable replication	Session structure
9	Detailed description of any home programme component	Exercise programme rationale
10	Describe whether there are any non-exercise components	Control group
11	Describe the type and number of adverse events that occur during exercise	Exercise programme characteristics
12	Describe the setting in which the exercises are performed	Exercise programme characteristics
13	Detailed description of the exercise intervention	Intervention
14a	Describe whether the exercises are generic (one size fits all) or tailored	Exercise programme characteristics
14b	Detailed description of how exercises are tailored to the individual	Exercise programme characteristics
15	Describe the decision rule for determining the starting level	Exercise programme rationale
16a	Describe how adherence or fidelity is assessed/measured	Participant adherence and compliance
16b	Describe the extent to which intervention was delivered as planned	Participant adherence and compliance

Since plyometric jump training has been shown to be effective in improving bone health and to maintain the benefits after the intervention in children and adolescents [35], jumping exercise will be the basis for the specific exercise type in iBoneFIT. Notwithstanding, the Exercise Guidelines for Cancer Survivors recommend an extended phase of resistance training before progressing to impact loading [44]. In this sense, a recent systematic review highlighted that resistance training should be incorporated at an early age and prior to plyometric training in order to establish an adequate foundation of strength for power training activities [47]. Therefore, all participants will start with a familiarisation phase aimed to improve muscular fitness before implementing mechanical loading through jumps (Item 7a and 15).

Although the duration of the jumping interventions to be effective on bone outcomes in children and adolescents is unclear, the length of the exercise programme will be 9 months based on results from previous studies [38, 48]. In addition, we have considered the fact that bone remodelling process requires approximately 5 months [49]. Dietary counselling on calcium and vitamin D will be provided to the participants in both control and intervention groups due to having an adequate calcium and vitamin D levels is important as both interact with physical activity to enhance bone mass (Item 9) [28, 50].

#### Exercise programme characteristics

This home-based intervention will be delivered online by making use of social media (Item 4 and 12). Using popular existing social network sites may address issues of reach, engagement, and retention [51, 52]. WhatsApp (WhatsApp Inc., Mountain View, CA, USA) is a highly used app in Spain for social networking and that allows us to send text messages and other types of media (e.g. photos and videos) to the parents of participants. Although WhatsApp has been revealed as a feasible method to deliver exercise interventions, Muntaner-Mas et al. [53] have suggested that the implementation of behaviour change techniques could increment the effectiveness on the outcomes assessed. Thus, five behaviour change techniques (i.e. action planning and goal setting, providing instructions and demonstrations of how to perform the behaviour, self-monitoring of behaviour, providing feedback on performance and information about health consequences) and a gamification design (i.e. points and rankings) will be included to improve the interest and incentive of this non-game programme (Table 2) (Item 6). These motivational approaches were chosen because of their known effect on physical fitness [53], physical activity [54] and satisfaction [55]. Moreover, parents will be told to encourage their children to perform the exercise programme in order to increase motivation.

**Table 2:** Translation and operationalization of BCTs targeting behaviour determinants into BIT elements

Determinant	BCT	Operationalization	BIT element	Workflow
Perceived behavioural control; Autonomy; Planning; knowledge/awareness	Action planning and goal setting (behaviour)	Inform the participants about the phase of the intervention and goals	WhatsApp group message	Every 2 weeks (Sunday)
Perceived behavioural control; Intentions; Competence; Knowledge/awareness	Provide instructions and demonstrations on how to perform the behaviour	Give instructions and demonstrations about how to perform the training session	Videos with exercise proposals	Every 2 weeks (Sunday)
Perceived behavioural control; Autonomy; Competence; Knowledge/awareness	Prompt self-monitoring of behaviour	Ask the participants to report the intervention compliance	WhatsApp group message	Every 2 weeks (Sunday)
Perceived behavioural control; Relatedness; Competence; Knoledge/awareness	Provide feedback on performance	Inform the participants about their performance in the main exercises (i.e. body mass-based squat, squat jump and countermovement jump)	WhatsApp group message or video	Every 2 weeks (Friday)
Perceived behavioural control; Attitude (beliefs); Knowledge/awareness	Information about health consequences	Present press releases to emphasize the importance of calcium and vitamin D for bone health	WhatsApp group message	At the beginning of each phase (Sunday)

*BCT* behaviour change technique, *BIT* behaviour intervention technology

A personal trainer with a BSc degree in Sport Sciences will develop all the sessions of this programme (Item 2, 14a and 14b). The personal trainer will record 18 exercise sessions and they will be uploaded in a private channel of the YouTube website. Each of them will be repeated over a 2 weeks period. The YouTube platform has been reported to be an educational tool for healthcare conditions among people coping with illness [56]. Every new session for the following 2 weeks will be shared through the WhatsApp group every 2 weeks. Finally, participants will perform the exercise programme individually or accompanied (i.e. with parents or friends) according to their preferences (Item 3) [57]. They will be required to record videos and send through the WhatsApp group in order to supervise the execution of the jumping exercises by the personal trainer. The exercise programme will be performed on a hard surface (Item 1) [58], and participants will be asked to report any pain or injuries at each stage of the intervention (Item 11).

#### Frequency and volume

Following the updated physical activity guidelines, children and adolescents should include bone-strengthening exercises as part of the daily physical activity on at least 3 days per week. Participants in the iBoneFIT study will perform the exercise programme three to 4 days per week (preferably on Mondays, Wednesdays and Fridays; or Mondays, Tuesdays, Thursdays and Fridays). If one training session is missed, the participant will be able to do it on a different day of the week, provided a minimum of 24 h of rest.

The total volume will be 7296 squat/jumps (2000 squats + 5296 jumps). The doses will be composed of 136 sessions (10–20 min/session) over 36 weeks. A full description of the training volume and its progression is shown in Table 3. In a recent 9-month RCT based on jumping activities with similar dosage we reached 70% of compliance (6216 jumps), and this was enough to improve bone outcomes in non-weight-bearing sport athletes [38]. Thus, the proposed volume of 7296 squat/jumps is likely to elicit the same effect in paediatric cancer survivors.

**Table 3:** iBoneFIT study exercise programme periodisation

Phase	Warm up^**a**^	Exercise^**b**^	Level	Repetitions	Sets a day (Rest^**c**^)	Sessions a Week	Squats/Jumps a Week
**1**	RAMP	BM-based Squat	1 (1–4 wk)	15	3	4	180
			2 (5–8 wk)	20	4	4	320
**Total phase 1 (8 wk)**							2000
**2**	RAMP	SJ	1 (9–12 wk)	10	3	3	90
			2 (13–16 wk)	15	3	4	180
			3 (17–20 wk)	20	4	4	320
**Total phase 2 (12 wk)**							2360
**3**	RAMP	CMJ	1 (21–24 wk)	10	3	3	90
			2 (25–28 wk)	12	3	4	144
			3 (29–32 wk)	15	3	4	180
			4 (33–36 wk)	20	4	4	320
**Total phase 3 (16 wk)**							2936
**Total intervention (36 wk)**							7296

*RAMP* raise, activate, mobilise and potentiate, *BM* body mass, *SJ* squat jump, *CMJ* countermovement jump

^a^Warm up will be focused on dynamic exercises with progressive intensity enhancing optimal core body temperature, motor unit excitability, kinesthetic awareness and ranges of motion

^b^Each exercise will be suggested to be performed at the pace of the personal trainer managing the session. If not, a self-paced performance will be recommend

^c^Phase 1 rest = 45 s

Phases 2 and 3 rest = 1 min

#### Periodisation

Although Peitz et al. [59] did not find differences between no, linear and undulating periodisations in youth, iBoneFIT will implement a linear model based on the fact that variation in volume and/or impact loading within the programme phases may stimulate greater bone adaptions and reduce boredom and risk of over-training [44]. The exercise programme will be divided in three phases of different durations and impact loadings (i.e. height reached in the different jumps). Each phase will be composed of levels with progressive increase in volume (i.e. repetitions, sets per day and sessions per week) as shown in Table 3 (Item 7b).

The phase 1 corresponds to the first 8 weeks of the exercise programme. Participants will perform body mass-based squats and the volume will increase progressively by modifying the number of repetitions and sets per day. Paediatric cancer survivors may present reduced aBMD and muscular fitness [60], therefore jumping exercise prescription may not be safe. In this sense, body mass-based squat was chosen in this phase following previous studies that observed positive effects on muscular fitness after an 8-week intervention [61, 62].

The phase 2 will last 12 weeks and participants will perform squat jumps. In this phase, the volume will increase progressively by modifying the number of repetitions, sets per day and sessions per week. Squat jump has been chosen as intermediary exercise before the use of countermovement jump since the jump height reached is lower and hence, ground reaction forces produced at the landing are lower [63]. Furthermore, squat jump training reduces the degree of muscle slack on the push-off phase [64] which could supply a better execution of the countermovement jump afterwards.

The phase 3 will be the longest phase of the exercise programme with 16 weeks. Participants will perform countermovement jumps and the volume of this phase will be increased progressively by modifying the number of repetitions, sets per day and sessions per week. Countermovement jump will be chosen in this phase since it produces a huge force application (~ 400 times body mass / second) and ground reaction forces (~ 5 times body mass) in youth [65, 66]. Countermovement jump has been previously reported to be valid and reliable in children [67].

#### Session structure

The structure of the exercise sessions will be: 1) warm up; 2) squat/jumps training; 3) cool down. Briefly, the warm ups will be based on RAMP methodology (i.e. raise, activate, mobilize and potentiate) in order to maximize middle-term performance of the main exercises (i.e. squat/jumps exercises) [68]. Eight exercises focused on the brace, squat, lunge or jump patterns will be included in this part of the session. Squat/jumps training will comprise body mass-based squats, squat jumps and countermovement jumps in phase 1, phase 2 and phase 3, respectively. Finally, participants will perform a cool down including static stretching and relaxing exercises (Item 8).

### Control group

Participants randomly allocated to the CG will receive information on the recommendations of calcium and vitamin D [69]. Educational leaflets and infographics based on the current recommendations [69] will be delivered to the participants at the beginning of the study (Item 10). After finishing the study, they will be offered the same online exercise programme.

## Outcomes

The primary outcome of our study is bone health. The secondary outcomes include anthropometric measurements, body composition, physical fitness components, free-living physical activity, blood samples, calcium and vitamin D intake, health-related quality of life and mental health. Assessments will be conducted at baseline, repeated at post-test (i.e. after 2 weeks of intervention or control condition at most) and follow up (i.e. after 4 months of intervention or control condition). Participants will be assessed for the post-test and follow up following the order through which they will be tested at baseline, to avoid cofounding by time between baseline and the other assessments.

Data obtained on the assessments will be recorded on a paper print-out and entered into an Excel file for future statistical analysis. Questionnaires will be filled using Google Forms which allows us to record the data without hand-written management. In compliance with the Personal Information Protection Act, the names of all participants will not be disclosed, and an identifier number will be used to identify each participant. All participants will be informed that the clinical data obtained in the trial will be stored in a computer and will be handled with confidentiality.

### Primary outcome: bone health

#### Dual-energy X-ray Absorptiometry (DXA)

A DXA (Hologic Series Discovery QDR, Bedford, MA, USA) will be used throughout the study to obtain BMC (g) and aBMD (g/cm^2^) for the hip, lumbar spine and total body less head. Furthermore, lean soft tissue mass (g), fat mass (kg) and body fat percentage (%) for the whole body will be obtained from total body scans. APEX software (version 4.0.2) will be used to analyse the scans following the recommendations for children and adolescents [70]. Equipment calibration, participant setting and scan analyses will be performed by the same researcher. DXA uses a minimal radiation (i.e. spending a day outside in the sunshine) and the effective dose for the scans in children has been set in 3–6 μSv [71].

##### Hip Structural Analysis (HSA)

HSA is a DXA-based software that analyses hip scans to estimate bone geometric properties of the proximal femur. This software analyses structural characteristics through the distribution of bone mineral mass in a line of pixels across the bone axis [72]. These geometric estimates in the proximal femur will be derived from: 1) the cross-sectional area (mm^2^); 2) section modulus (mm^3^); and 3) the cross-sectional moment of inertia (mm^4^). For these variables, the short-term coefficient of variation has been reported to be between 2.4 and 10.1% [73].

##### Trabecular Bone Score (TBS)

TBS is a DXA-based software (iNsight version 3.0, Medimaps, Pessac, France) that indirectly assesses the state of trabecular microarchitecture in the lumbar spine. Based on experimental variograms of the projected DXA image, TBS evaluates the heterogeneity of the grey-levels pixels of the aBMD and higher heterogeneity implies worse trabecular connectivity [74]. Low values reported in this parameter have been associated with a higher fracture risk, and therefore it is considered an index of bone quality [75]. The short-term coefficient of variation for TBS has been reported to be between 1.7 and 2.1% for spine aBMD in 92 individuals with repeated spine DXA scans performed within 28 days [76].

##### 3D-DXA Modelling

3D-SHAPER is a DXA-based software (version 2.2, Galgo Medical, Barcelona, Spain) that derives 3D analyses from the hip DXA scans. Details of the model algorithm are published elsewhere [77]. Briefly, this software uses a 3D statistical shape and density of the proximal femur built from a database of quantitative computed tomography (QCT) scans of Caucasian population [77]. The 3D-SHAPER will assess bone parameters such as the cortex, the femoral shape and the trabecular macrostructure [78].

The cortex is segmented by fitting a mathematical function of the cortical thickness (mm), cortical volumetric BMD (cortical vBMD, mg/cm^3^), the location of the cortex, the density of surrounding tissues and the imaging blur to the density profile computed along the normal vector at each node of the proximal femur surface mesh [78]. In addition, the cortical surface BMD (cortical sBMD, mg/cm^2^) is computed at each vertex of the femoral surface mesh, as the multiplication of the cortical thickness (cm) by the cortical vBMD along its thickness [79]. Any increase in either cortical thickness or cortical vBMD will ensure an increase in cortical sBMD. Nevertheless, if cortical thickness and cortical vBMD vary in opposite ways, cortical sBMD will remain unchanged. All measurements will be computed over the total femur (i.e. the shaft, the intertrochanteric and the union of the neck) according to the trabecular, cortical and integral compartments.

Correlation coefficients between BMD computed by 3D-SHAPER and QCT of the total femur have been reported to be 0.86–0.95, whereas the correlation coefficients of BMD computed by 3D-SHAPER with BMD computed by QCT have been reported to be 0.91 [77]. The short-term coefficients of variations of aBMD measurements have been reported to be 1.5, 4.5, 1.7 and 1.5% for cortical thickness, trabecular vBMD, cortical vBMD and cortical sBMD, respectively [79].

### Secondary outcomes

#### Anthropometric measurement, body composition and somatic maturation

Body mass (kg) will be measured with an electronic scale (SECA 861, Hamburg, Germany) with an accuracy of 100 g. Height (cm) will be measured by using a precision stadiometer (SECA 225, Hamburg, Germany) to the nearest 0.1 cm. Body mass index (BMI) will be calculated as body mass (kg)/height (m^2^), and the participants will be classified into BMI categories according to sex- and age-specific cut offs [80].

In addition to DXA measurements, a bioimpedance scale (Tanita BC-418 MA; Amsterdam, The Netherlands; range: 2–200 kg; precision: 0.1 kg; body fat percentage increments: 0.1%) will estimate the percentage of body fat of the participants. The assessment will be carried out in fasting state according to the manufacturer’s instructions. Despite the measured error, bioelectrical impedance analysis will be used to assess body fat as it is considered a practical method in addition to DXA [81].

Somatic maturation will be assessed using the prediction of years from peak height velocity using validated algorithms for children [82].

#### Physical fitness

The ALPHA fitness test battery will be used to assess physical fitness. These field-based fitness tests have been shown to be valid, reliable and related to health in children and adolescents [83]. In brief, cardiorespiratory fitness will be assessed with the 20 m shuttle run test; muscular fitness will be assessed with the handgrip strength and standing long jump tests; and speed agility will be assessed with the 4 × 10 m shuttle run test. All tests will be performed twice, and the best score will be retained, except 20 m shuttle run test.

Perceived physical fitness will be assessed by the International Fitness Scale (IFIS). The IFIS is a short, simple and self-administered scale that has been validated in children and adolescents [84, 85]. This 5-item scale asks the participants about their physical fitness comparing with their colleagues.

#### Physical activity and sedentarism

Physical activity and sedentary behaviours will be objectively assessed at the baseline, post-intervention and follow-up measurements. Participants will wear a tri-axial accelerometer (ActiGraph GT3X, Pensacola, FL, USA) attached to the non-dominant wrist over seven consecutive days (24 h/day) and they will remove it only for water-based activities (e.g. bathing or swimming). They will also have a diary in order to record the time when they go to bed, wake up and remove the device. Correlation coefficient between accelerometer measured metabolic energy equivalents and indirect calorimetry has been reported to be 0.65 [86], whilst correlation coefficient of accelerometer impact loading and ground reaction forces by force platforms has been reported to be 0.74 [87].

In addition, information on self-reported physical activity and sedentary behaviours will be obtained by the cross-translated and adapted version of the Youth Activity Profile (YAP) questionnaire (available at: http://profith.ugr.es/yap?lang=en). The YAP questionnaire was developed at the Iowa State University and validated in children [88]. This self-administered 7-day recall questionnaire collects data from items regarding physical activity in the school setting, physical activity out of the school setting, activity immediately after school, activity during the evening and activity during each weekend day. Moreover, the bone-specific physical activity questionnaire (BPAQ) will be used to assess the influence of historical physical activity (i.e. activities in which you have ever participated, and activities practiced in the last 12 months) on skeletal health. It has been reported that BPAQ is a valid instrument to account for the effects of previous physical activities on the skeleton [66].

#### Calcium intake and vitamin D status

To correctly interpret bone health of the participants, an assessment of dietary intake of calcium will be completed at the baseline, post-intervention and follow-up measurements. A validated food-frequency questionnaire will be used to estimate calcium intake [89]. In addition to plasma 25-hydroxyvitamin D levels obtained from blood analyses (see blood samples section), a vitamin D questionnaire to assess the status of this prohormone will be implemented [90].

#### Blood samples

Fasting blood samples will be collected by venepuncture between 8:00 and 10:00 after an overnight fast. The methodology for shipment, preparation and collection of the blood samples was standardized among all participating hospitals. A set of parameters obtained from haematological and biochemical analyses will be available from the hospitals as part of the follow-up protocols.

#### Health-related quality of life and mental health

The Paediatric Quality of Life Inventory (PedsQL™ 4.0 Generic Core Scales) will be used to assess quality of life. PedsQL™ is validated in paediatric cancer survivors and has been successfully used [91]. This 23-item scale assesses quality of life considering five domains of health (i.e. physical functioning, emotional functioning, psychosocial functioning, social functioning and school functioning). Results from our participants in all domains of PedsQL™ will be compared to published normative data [92].

Childhood anxiety will be assessed with the State-Trait Anxiety Inventory for Children (STAIC-T). This inventory has been extensively validated in Spanish children [93]. Depression will be measured with the Children Depression Inventory (CDI), which consists of 27 items that assesses 5 domains (interpersonal problems, ineffectiveness, negative mood, anhedonia and negative self-esteem) [94]. Rosenberg Self-Esteem scale will be used to assess self-esteem and has been validated with children and adolescents [95]. We will use the Positive Affect Schedule for children (PANAS-C) in order to measure both positive and negative affect [96]. The original PANAS-C reported appropriate values of internal consistency (0.86 for the positive affect and 0.82 for the negative affect). Happiness will be assessed by the Subjective Happiness Scale (SHS) whose Spanish version has shown appropriate test-retest reliability, internal consistency and convergent validity [97]. Dispositional optimism will be assessed with the Life Orientation Test-Revised (LOT-R) [98]. LOT-R is an instrument with good internal consistency (0.71 for the total score and of 0.64 and 0.77 for the optimism and pessimism, respectively) [99].

## Discussion

The iBoneFIT study will examine the effect of an online RCT exercise programme on bone health in paediatric cancer survivors aged 6–18 years old. In addition, it will follow up the participants 4 months after the intervention to examine whether the effects remain. Finally, the iBoneFIT study will investigate whether the intervention affects body composition, physical fitness, physical activity, quality of life and mental health of paediatric cancer survivors.

Previous evidence shows a higher risk of delayed bone development, diminished muscle functioning, disability and compromised fundamental movement skill acquisition in children who have completed cancer treatment [79]. These side effects can also reduce motivation to be physically active and aggravate chronic health conditions in the short and long terms [100]. A recent longitudinal review showed that higher levels of muscular fitness in childhood and adolescent were associated with higher aBMD later in life [101]. Moreover, jumping interventions have shown improvements in both muscular fitness and bone health [38, 39, 102]. In this regard, Vlachopoulos et al. [39] reported an increase in physical fitness (3.7–7.9%) and BMC at TBLH and legs (4.2–12.7%) in non-osteogenic sports. Additionally, Vlachopoulos et al. [38] showed that a 9-month plyometric training improved LS BMC (4.6%) and femoral neck BMC (6–9.8%) in non-osteogenic sports. Another study carried out by Mackelvie et al. [102] suggested that jump training was associated with increases in femoral neck, lumbar spine and total body aBMD (~ 2%) in prepubertal boys and may delay the onset of osteoporosis later in life. The Exercise Guidelines for Cancer Survivors recommend avoiding movements that place excessively high load on fragile skeletal sites [43]. Thus, our intervention aims to improve muscular strength before implementing mechanical loading through jumping exercises.

Several studies highlight that exercise interventions delivered during cancer treatment are not successful in improving bone health nor other factors such as health-related quality of life [40, 42], suggesting a new approach focusing on post treatment phase is needed. Furthermore, following the study of McKay et al. [36], the exercise programme should be effective, possible to perform at any place, short in duration, inexpensive and simple to administer. iBoneFIT has been designed to meet all these requirements. The exercise programme will be delivered online and using social media which also guarantees social distancing nowadays. Some international physical activity interventions based on online and app-based approaches have shown promising results, indicating the suitability of this technology to influence health behaviours [103, 104]. Therefore, analysing the effect of this exercise programme in paediatric cancer survivors is of scientific interest.

iBoneFIT represents a golden opportunity to analyse for the first time the effect of a simple, feasible, inexpensive and short duration exercise programme on bone health in paediatric cancer survivors. This study will target this population in high risk of low bone mass, using an enjoyable intervention and cutting-edge technologies (i.e. DXA and tri-axial accelerometers) to assess its effectiveness. If successful, this 9-month online exercise program will likely encourage paediatric cancer survivors to be physically active or even engage in a sport, providing an opportunity to decrease chronic health conditions in the short and long terms [6]. Finally, iBoneFIT will substantially contribute to the existing knowledge of how physical activity affects quality of life and mental health in this population. The long-term medical and psychological effects of childhood cancer or its treatment may negatively affect social functioning such as school attendance, obtaining employment and even social activities [100]. Therefore, their quality of life and mental health are important concerns, and efforts have to be made to improve it, which will have an important societal and economic impact.

## Data Availability

The data that will be generated and analysed during the current study is not publicly available due to the sensitivity of the collected data. The data are available from the corresponding author upon reasonable request.

## Abbreviations

BMD: Bone mineral density
BMC: Bone mineral content
aBMD: Areal bone mineral density
vBMD: Volumetric bone mineral density
sBMD: Surface bone mineral density
RCT: Randomized controlled trial
IG: Intervention group
CG: Control group
CERT: Consensus on exercise reporting template
BCT: Behaviour change techniques
BIT: Behaviour intervention technology
DXA: Dual-energy x-ray absorptiometry
HSA: Hip structural analysis
TBS: Trabecular bone score
BIA: Bioimpedance analysis
IFIS: International fitness scale
YAP-S: Youth activity profile-Spain
BPAQ: Bone-specific physical activity questionnaire
PedsQL: Paediatric quality of life inventory
STAIC-T: State-trait anxiety inventory for children
CDI: Children depression inventory
PANAS-C: Positive affect schedule for children
SHS: Subjective happiness scale
LOT-R: Life orientation test-revised
